# Understanding Whether and How a Digital Health Intervention Improves Transition Care for Emerging Adults Living With Type 1 Diabetes: Protocol for a Mixed Methods Realist Evaluation

**DOI:** 10.2196/46115

**Published:** 2023-09-13

**Authors:** Ruoxi Wang, Geneviève Rouleau, Gillian Lynn Booth, Anne-Sophie Brazeau, Noor El-Dassouki, Madison Taylor, Joseph A Cafazzo, Marley Greenberg, Meranda Nakhla, Rayzel Shulman, Laura Desveaux

**Affiliations:** 1 Institute for Better Health Trillium Health Partners Mississauga, ON Canada; 2 Institute for Health System Solutions and Virtual Care Women’s College Hospital Toronto, ON Canada; 3 Département des Sciences Infirmières Université du Québec en Outaouais St-Jérôme, QC Canada; 4 Faculté des sciences infirmières l'Université de Montréal Montreal, QC Canada; 5 MAP Centre for Urban Health Solutions Unity Health Toronto Toronto, ON Canada; 6 Department of Medicine University of Toronto Toronto, ON Canada; 7 Institute of Health Policy, Management and Evaluation University of Toronto Toronto, ON Canada; 8 School of Human Nutrition McGill University Montréal, QC Canada; 9 Centre for Digital Therapeutics Toronto General Hospital University Health Network Toronto, ON Canada; 10 Institute of Biomedical Engineering University of Toronto Toronto, ON Canada; 11 Department of Computer Science University of Toronto Toronto, ON Canada; 12 Department of Philosophy Joint Centre for Bioethics University of Toronto Toronto, ON Canada; 13 Diabetes Action Canada Toronto, ON Canada; 14 Division of Endocrinology Montreal Children’s Hospital McGill University Montréal, QC Canada; 15 Research Institute of the McGill University Health Centre Montréal, QC Canada; 16 Child Health Evaluative Sciences SickKids Research Institute Toronto, ON Canada; 17 Division of Endocrinology The Hospital for Sick Children Toronto, ON Canada; 18 Department of Pediatrics University of Toronto Toronto, ON Canada

**Keywords:** digital health, emerging adults, realist evaluation, self-management, transition to adult care, type 1 diabetes

## Abstract

**Background:**

Emerging adults living with type 1 diabetes (T1D) face a series of challenges with self-management and decreased health system engagement, leading to an increased risk of acute complications and hospital admissions. Effective and scalable strategies are needed to support this population to transfer seamlessly from pediatric to adult care with sufficient self-management capability. While digital health interventions for T1D self-management are a promising strategy, it remains unclear which elements work, how, and for which groups of individuals.

**Objective:**

This study aims to evaluate the design and implementation of a multicomponent SMS text message–based digital health intervention to support emerging adults living with T1D in real-world settings. The objectives are to identify the intervention components and associated mechanisms that support user engagement and T1D health care transition experiences and determine the individual characteristics that influence the implementation process.

**Methods:**

We used a realist evaluation embedded alongside a randomized controlled trial, which uses a sequential mixed methods design to analyze data from multiple sources, including intervention usage data, patient-reported outcomes, and realist interviews. In step 1, we conducted a document analysis to develop a program theory that outlines the hypothesized relationships among “individual-level contextual factors, intervention components and features, mechanisms, and outcomes,” with special attention paid to user engagement. Among them, intervention components and features depict 10 core characteristics such as transition support information, problem-solving information, and real-time interactivity. The proximal outcomes of interest include user engagement, self-efficacy, and negative emotions, whereas the distal outcomes of interest include transition readiness, self-blood glucose monitoring behaviors, and blood glucose. In step 2, we plan to conduct semistructured realist interviews with the randomized controlled trial’s intervention-arm participants to test the hypothesized “context-intervention-mechanism-outcome” configurations. In step 3, we plan to triangulate all sources of data using a coincidence analysis to identify the necessary combinations of factors that determine whether and how the desired outcomes are achieved and use these insights to consolidate the program theory.

**Results:**

For step 1 analysis, we have developed the initial program theory and the corresponding data collection plan. For step 2 analysis, participant enrollment for the randomized controlled trial started in January 2023. Participant enrollment for this realist evaluation was anticipated to start in July 2023 and continue until we reached thematic saturation or achieved informational power.

**Conclusions:**

Beyond contributing to knowledge on the multiple pathways that lead to successful engagement with a digital health intervention as well as target outcomes in T1D care transitions, embedding the realist evaluation alongside the trial may inform real-time intervention refinement to improve user engagement and transition experiences. The knowledge gained from this study may inform the design, implementation, and evaluation of future digital health interventions that aim to improve transition experiences.

**International Registered Report Identifier (IRRID):**

PRR1-10.2196/46115

## Introduction

Type 1 diabetes (T1D) is a common chronic condition that affects 9 million people globally [1], including over 650,000 children (aged 0-14 years) and 560,000 teenagers (aged 15-19 years) [2]. T1D management requires knowledge, skills, and motivation to perform daily self-management as well as routine health care management and monitoring throughout the lifespan [3-5]. A total of 42% of individuals with T1D onset during childhood often experience deterioration in glycemic management, which confers an increased risk of chronic complications and acute diabetes complications such as life-threatening ketoacidosis during adolescence and early adulthood [1,6]. Effective health care management and a smooth transition from pediatric to adult care can help mitigate this risk [5,7,8]. Unfortunately, the transition period is fraught with challenges in part because it coincides with emerging adulthood, when individuals are facing a series of changes in their independence and responsibilities for disease management [9]. Current estimates suggest that over 20% of emerging adults experience a gap of over 6 months between pediatric and adult care medical visits during transition [10,11]. Effective transition support must attend to medical, psychological, educational, and vocational needs in order to support sufficient self-management skills [12-14]. Failure to address these needs may lead to adverse health outcomes [14], decreased health system engagement [10], and increased hospital admissions [15].

Strategies to improve the transition process have been implemented at the patient, provider, and service levels [16,17], but are often institution-specific or resource-intensive, thereby limiting their accessibility, scalability, and generalizability [18-20]. Digital health interventions are increasingly used to address these limitations while also leveraging the high prevalence of smartphone use among youth [21]. The hypothesized benefits are also compelling, including the flexibility to access and exchange information instantly, irrespective of geographic constraints, at low cost, and the potential to provide more personalized support [22]. Digital health T1D management interventions, through mobile apps [23-25], websites [26], and text messages, emails, or telephone calls [25,27-29], have become increasingly common [23-29] and acceptable [23,26,27] to young adults, with demonstrated impacts on clinic attendance [28] and glycemic control [29]. A recent paper by Cafazzo et al [23] piloting a T1D management mobile app in adolescents found 88% of participants were satisfied with the app. Moreover, they found a significant increase in the frequency of blood glucose measurement among their participant sample. In a pragmatic clinical trial, Butalia et al [28] observed significantly greater outpatient appointment attendance in transitioning youth living with T1D who received a communication technology-based (SMS text message, email, or telephone) transition coordinator intervention as compared to those who received care as usual. Also, through a crossover trial with adolescents, Rami et al [29] found SMS text messaging-based telemedical support feasible and helpful in improving hemoglobin A_1c_ (HbA_1c_).

However, potential impact can be undermined by the often-reported rapid drop in intervention engagement, which occurs sometime between 2 weeks and 6 months after initial use [30,31]. Among adolescents living with T1D exposed to a mobile self-management tool, 1 study reported that only 35% of participants were either moderately or highly engaged over the 12-month intervention [24]. Despite this well-known risk, comparatively little is known about how to overcome this common challenge. Relatedly, many multicomponent digital health interventions report aggregate effects [27-29], limiting our ability to understand which elements contribute to achieving the distinct but related goals of ensuring sustained intervention engagement while also effectively addressing the known barriers to desired outcomes.

Realist evaluation, a theory-driven evaluation framework [32], seeks to unpack the black box of interventions by establishing the causal links between intervention resources and associated outcomes while identifying the circumstances needed to facilitate the change in a Context-Mechanism-Outcome (CMO) framework [33-36]. Given the complex and context-specific nature of health interventions [37], they have been increasingly adopted in the health field [34,38-40] to solve the question of what works, for whom, and in what circumstances [41-43].

By acknowledging the potential of realist evaluation in addressing the abovementioned knowledge gaps, we have developed a protocol for a sequential mixed methods realist evaluation embedded alongside a randomized controlled trial (RCT) evaluating the effect of a digital health intervention on the transition experiences of emerging adults living with T1D. Using a realist evaluation framework, the specific objectives of the embedded realist evaluation will be to (1) develop and test the intervention program theory that identifies what elements of the intervention contribute to successful engagement and improve transition experiences, how they do it, and for which groups of individuals; and (2) consolidate the intervention program theory by identifying the crucial combinations of factors that are minimally sufficient for an effective implementation of the digital health intervention and their coexistent causal pathways.

## Methods

### Study Design

**Figure 1:**
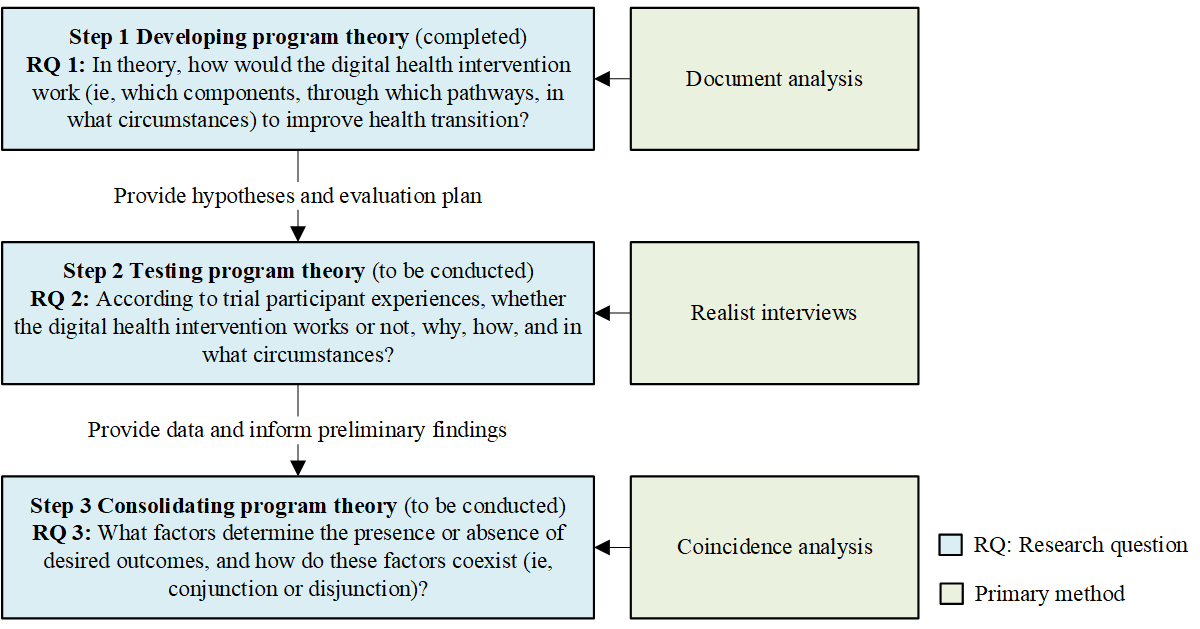
Study design and methods.

Following Mirzoev et al [40], we plan to conduct this embedded realist evaluation in 3 steps (Figure 1), which include developing (step 1; completed), testing (step 2; to be conducted), and consolidating the program theory (step 3; to be conducted).

As a theory-based evaluation and in line with best practices [32,44], this study is guided by a program theory outlining the hypothesized mechanisms through which the digital health intervention is expected to work and the corresponding context, with specific attention paid to user engagement (step 1). We have added an explanatory factor “intervention” (I) to the conventional “context-mechanism-outcome” (CMO) framework [45] following Breton et al [46] and Shams et al [47] to disaggregate the 2 major components of mechanisms (M) in the conventional CMO framework (ie, intervention resources and recipients’ reasoning) [36]. This will enable us to specify which elements of this multicomponent intervention contribute to the desired outcomes and how they are influenced by context [44]. Specifically, we have developed the initial program theory in the framework of “context-intervention-mechanism-outcome” (CIMO) to understand for individuals with which contextual characteristics (C), which intervention components and features (I), trigger what mechanisms (M), and therefore yield what target outcomes (O). This initial program theory informs the following data collection and analysis [39].

Since realist evaluation is method neutral [48], this study uses mixed methods and integrates multisource data to test and consolidate the program theory. In step 2, we plan to conduct semistructured realist interviews with participants in the RCT intervention arm in a “teacher-learner cycle” [49]. The interviews will aim to test each of the hypothesized CIMO configurations by exploring individual perspectives and experiences using “Keeping in Touch” (KiT), including how participants interact with KiT and under what circumstances it facilitates (or fails to facilitate) mechanisms that lead to engagement and improved transition experiences.

Multicomponent interventions often achieve desired outcomes through distinct combinations of factors that interact to produce the outcome, as well as through multiple mechanisms [38]. For instance, the combination of a high level of positive outcome expectancies (ie, belief about the consequence of performing a specific behavior) and a high level of self-efficacy (ie, confidence about the ability to perform a specific behavior) may lead to improved diabetes self-care [50,51], whereas the combination of a high level of outcome expectancies and a low level of self-efficacy may lead to poorer diabetes self-care [51]. This highlights the need to identify difference-making factors as well as the settings in which they make a difference. Coincidence analysis (CNA) is a novel configurational approach underpinned by Boolean algebra to identify the crucial difference-making conditions, which include combinations of factors (including contextual factors and intervention components) necessary to achieve target outcomes and the causal pathways (mechanisms) that lead to positive effects [52]. Therefore, in step 3, we will leverage intervention usage data, patient-reported outcomes, and interview data and perform CNA to gain such an in-depth understanding, according to which we will consolidate the program theory.

### Step 1: Developing Program Theory

#### Document Analysis

We conducted a document analysis to develop the program theory using 2 data sources. First, we reviewed the program documentation, including the intervention design documents, RCT protocol, and data collection materials, to identify its intervention components, extract the corresponding features, understand the assumptions of the designers on how this multicomponent digital health intervention would lead to desired outcomes, and select the corresponding outcome indicators. Second, we reviewed published research articles on behavioral science theories, with special attention paid to digital behavior change interventions (DBCIs) and chronic disease self-management. The objective for reviewing pre-existing theories was twofold: (1) to inform the coding of intervention features, determine the key mechanisms, and identify the individual characteristics that may influence the implementation process; and (2) to select appropriate preexisting conceptual frameworks to inform the development of CIMO configurations, that is, build causal relationships among the constructs [53]. We focused on microlevel theories according to the study objective, that is, to understand the implementation process of a digital health intervention among individuals.

#### Intervention Components and Features

The KiT intervention [20] was developed using a user-centered design approach that engaged adolescents and emerging adults living with T1D as well as adult and pediatric diabetes providers. Specific intervention content was informed by clinician consultation and an environmental scan of diabetes transition resources mapped to domains of the “readiness of emerging adults with diabetes diagnosed in youth” (READDY) tool with the aim of improving the transition experiences among emerging adults living with T1D (Textbox 1). In addition to providing care coordination support (eg, appointment note-keeping and reminders), KiT is designed to provide personalized T1D informational support (eg, educational content and a question and answer feature) based on an individual’s interests and self-reported confidence about their diabetes knowledge and skills using a set of “if-then” rules (Multimedia Appendix 1).

**Brief description of the digital intervention.**

**Intervention name:** Keeping in Touch (KiT)

**Delivery method**: SMS chatbot

**Study population**: Emerging adults living with type 1 diabetes (T1D) residing in Ontario or Quebec who are within 4 months of their planned transfer to adult care

**Intervention length**: 12 months

**Intervention development methods:** Based on user-centered design approaches (detailed information can be found [54])

**Intervention components**:

- Informational content (one topic per month):
  - Topics received by all users: 4 topics provided to all participants at months 1, 4, 7, and 10 respectively. Topics include coping with T1D, care navigation, sick day and ketone management, medical insurance and financial support.
  - Topics based on user needs and interests: 8 topics from a pool of 10 candidate topics provided at the rest of 8 months. Topics are determined by the participant’s baseline transition readiness status (self-reported confidence about diabetes knowledge and skills measured by the “readiness of emerging adults with diabetes diagnosed in youth” READDY tool) and their interests. Topics include hypoglycemia, pumps and pump programming, insulin adjustments, drugs and alcohol, travel, driving, school and work accommodations, exercise, nutrition and carbohydrates, and sexual health.
- **Question and Answer**: KiT recognizes key words in participants’ T1D-related questions and automatically pulls resources from the KiT database to answer their question. Interaction with participants by periodically asking for feedback 5 minutes after providing question and answer responses.
- **Transition care coordination:**
  - Reminder: KiT sends reminders for participants to book appointments and requests them to input their appointment information in the chatbot. KiT then sends appointment and bloodwork or urine test reminders at the participant’s preferred times.
  - Note-keeping: KiT allows participants to save a list of items to discuss in their appointments, which is sent back to them 1 hour before their scheduled appointments.
  - Care coordination support: KiT sends prompts to help participants prepare for appointments and reflect on their care experiences.
  - Clinic information: KiT sends information on the adult clinic that the participant will be attending, for clinics included within the chatbot database.

We have identified intervention components and features (Table 1). By performing deductive coding according to existing literature, we extracted several common components and features of DBCIs, including personalization [55,56], problem-solving support [57-59], reminders [55], real-time interactivity [55,60], credible sources [57], user-friendly SMS text message tone [55], and diverse forms of information (enhanced media) [56]. We classified the remaining KiT components (eg, coping with T1D and care navigation) through inductive coding.

**Table 1:** Keeping in Touch (KiT) intervention components and features.

Intervention strategy and content				Timeline (month)		Component or feature
**Informational content**						
	**Topics received by all users**					
		Coping with T1D	1		Stress management strategies^a^	
		Care navigation	4		Transition support information^a^	
		Sick day and ketone management	7		T1D self-management information and suggestions^a^	
		Medical insurance and financial support	10		T1D self-management information and suggestions^a^	
	**Topics based on user needs and interests (selected by baseline readiness status and personal preferences [once every 3 months])**					
		Hypoglycemia	2, 3, 5, 6, 8, 9, 11, 12		T1D self-management information and suggestions^a^ Personalization^b^ [55,56]	
		Pumps and pump programming	2, 3, 5, 6, 8, 9, 11, 12		T1D self-management information and suggestions^a^	
		Insulin adjustments	2, 3, 5, 6, 8, 9, 11, 12		Personalization^b^ [55,56]	
		Drugs and alcohol	2, 3, 5, 6, 8, 9, 11, 12		T1D self-management information and suggestions^a^	
		Travel	2, 3, 5, 6, 8, 9, 11, 12		Personalization^b^ [55,56]	
		Driving	2, 3, 5, 6, 8, 9, 11, 12		T1D self-management information and suggestions^a^	
		School and work accommodations	2, 3, 5, 6, 8, 9, 11, 12		Personalizationb [55,56]	
		Exercise	2, 3, 5, 6, 8, 9, 11, 12		T1D self-management information and suggestions^a^	
		Nutrition and carbohydrates	2, 3, 5, 6, 8, 9, 11, 12		Personalization^b^ [55,56]	
		Sexual health	2, 3, 5, 6, 8, 9, 11, 12		T1D self-management information and suggestions^a^	
**Question and answer**						
	T1D self-management knowledge chatbot		1-12		T1D self-management information and suggestions^a^ Problem-solving support^a^ [57-59] Real-time interactivity^b^ [55,60]	
**Transition care coordination**						
	Reminders		1-12		Transition reminders^a^ [55]	
	Note-keeping		1-12			
	Care coordination support		1-12		Transition support informationa Personalization^b^ [55,56]	
	Clinic information		1-12		Transition support information^a^ Personalization^b^ [55,56]	
**Message content**						
	Information topics and resources were selected based on credibility		—^c^		Credible sources^b^ [57]	
	Young-adult friendly educational text messages		—		User-friendly message tone^b^ [55]	
**Message format**						
	Educational information in forms including but not limited to text, graphics, images, and videos		—		Diverse forms of information (enhanced media)^b^ [56]	
**Settings**						
	Frequency of receiving education content		—		Personalization^b^ [55,56]	
	Time of receiving messages		—		Personalization^b^ [55,56]	
	Times of receiving appointment reminders		—		Personalization^b^ [55,56]	
	Option to take a break from educational messages for 2 weeks (maximum 2 times throughout the intervention)		—		Personalization^a^ [55,56]	

^a^Intervention component.

^b^Intervention feature.

^c^Not available.

#### Initial Program Theory

**Figure 2:**
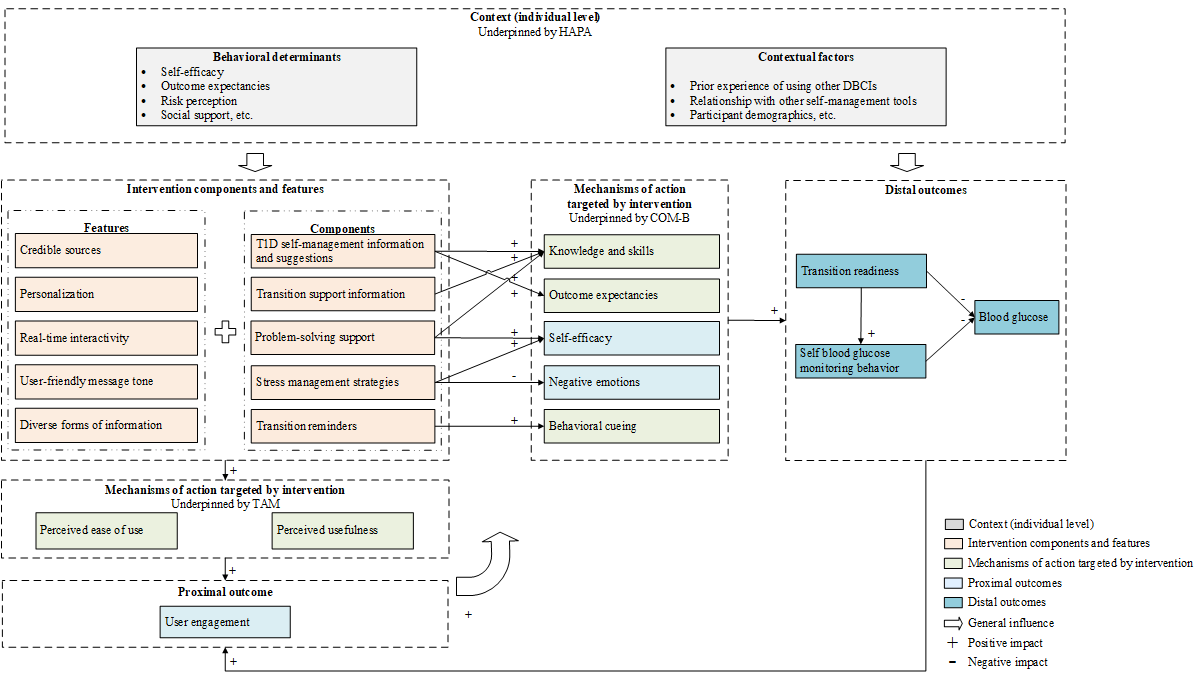
Initial program theory of the Keeping in Touch (KiT) intervention. COM-B: capability, opportunity, motivation, and behavior; DBCI: digital behavior change interventions; HAPA: health action process approach; T1D: type 1 diabetes; TAM: technology acceptance model.

We have developed the initial program theory (Figure 2) by integrating 3 empirically validated individual-level behavioral change theories, including “capability, opportunity, motivation, and behavior” (COM-B) [61], “health action process approach” (HAPA) [62], and “technology acceptance model” (TAM) [63].

Informed by COM-B [61], we hypothesize that KiT may improve participants’ transition outcomes (ie, transition readiness, self-management behavior, and health outcomes) by triggering changes in individual motivation (ie, self-efficacy, negative emotions, outcome expectancies, behavioral cueing) and capability (ie, knowledge, skills). T1D self-management information and suggestions, transition support information, and problem-solving support may increase participants’ T1D and transition-related knowledge and skills [64], and therefore, capability [61]. T1D self-management information and suggestions [65] and problem-solving support [59] may improve one’s outcome expectancies and self-efficacy, respectively, which are the key motivational factors of an individual’s behavioral change [66]. One’s self-efficacy may also be improved by receiving stress management strategies [67]. Meanwhile, stress management strategies may lead to reduced negative emotions [58,67], which have been recognized as one of the most prominent motivational challenges to a successful T1D health care transition [14,68]. Transition reminders may function as behavior cueing to plan for and attend clinic appointments [69]. Well-designed intervention functions, such as personalization, credible sources, and diverse forms of information, may amplify the impact of KiT’s intervention content.

The extent to which KiT can trigger changes in health care transition capability and motivation is hypothesized to be dependent on the level of user engagement [38,55,70,71]. Positive intervention outcomes (eg, improved transition readiness) may in turn incentivize increased user engagement [55], suggesting a positive feedback loop. According to TAM [63], user engagement is influenced by 2 core factors, that is, perceived usefulness and perceived ease of use [72], which may be influenced by intervention content and technological functions [73].

Intervention effectiveness depends heavily on the interaction of the intervention with users and their context [55], yet there is a paucity of contextual insights in the DBCI literature, creating a gap in our understanding of which contextual factors influence the efficacy of intervention mechanisms in T1D and how [74]. According to the HAPA framework [62], behavioral determinants, such as self-efficacy [75], outcome expectancies [66,75], risk perception [65,76], and social support [66,77], impact both outcome and the intervention implementation process itself. Contextual factors, such as the complementary or substitute relationships between KiT and other conventional self-management tools (eg, handwritten methods) [38], may also predict user engagement alongside more traditional variables (ie, participant demographics [55,70]).

### Step 2: Testing Program Theory

#### Participants

Eligible participants will include English- or French-speaking emerging adults living with T1D enrolled in the RCT intervention arm who provided informed consent to be contacted for the embedded process evaluation at the time of enrollment in the RCT. We are targeting a conservative sample size of 25-30 participants for the interviews based on previous realist interviews and CNA studies [38,42,78,79].

To increase the representativeness of the interview sample, we will purposively select participants with desired and undesired proximal outcomes of user engagement. This will be achieved by identifying “high-engagers” and “low- to medium-engagers” according to the intervention usage data at month 3 of the RCT. As there is no standard classification or threshold of DBCI engagement, we have used early KiT intervention usage data to identify a feasible definition of engagement. Considering the data availability, we conservatively define “high-engagers” as those with a 100% response rate to question prompts, with all other participants classified as “low- to medium-engagers.” All low- to medium-engagers will be invited for an interview. Should resource constraints not allow us to interview all high engagers, we will purposively sample to balance representation by gender and site of recruitment. The iterative sampling will continue until we reach thematic saturation (ie, no new themes are identified in the multisource data analysis), or all interested participants have been interviewed.

#### Data Collection

To test the initial program theory developed in step 1, we will perform semistructured realist interviews in a “teacher-learner cycle” [49]. The interviews will start with some general questions about the participants’ experiences managing T1D and engaging with KiT [80,81]. Based on their level of engagement and responses, the interviewer will act as a teacher to introduce the candidate CIMO configurations to the participant for their comments. After learning about the CIMO configurations, the participant will then act as teacher to confirm, extend, or refute the hypothesized pathways with their own examples of how they reacted to specific intervention components or features, what they saw as influencing their decision-making, and how this was perceived to affect their outcomes [80,82]. For low- to medium-engagers, the interview topics will focus on CIMO configurations regarding user engagement. For high engagers, the interview topics will focus on CIMO configurations regarding user engagement as well as distal outcomes (ie, transition readiness, self-blood glucose monitoring, and blood glucose). The interview guides (Multimedia Appendix 2) will first be piloted to ensure a comprehensive assessment of all relevant CIMO constructs. Interviews will be conducted by PhD-trained research coordinators with qualitative interview training, previous experience conducting semistructured interviews, and in-depth knowledge of the initial program theory. They will also receive study-specific training from the senior study lead, who is an experienced qualitative researcher. Research coordinators will have no previous relationship with study participants or the T1D community. Interviews will be transcribed verbatim by an independent third party.

#### Data Analysis

Transcripts will be coded using MAXQDA, a software for interview data analysis, and analyzed using the principles of thematic analysis strategies [82,83]. Data will be deductively coded and mapped to the predefined CIMO constructs to reflect triads (eg, context + intervention outcome, intervention mechanism outcome) and tetrads (context + intervention mechanism outcome) that would confirm, extend, or refute the current CIMO configurations [84]. Open coding will be applied when themes are identified that do not fit within the definitions of predefined CIMO constructs. The findings will be compared against the hypotheses developed in step 1, with a label of “supported,” “refined,” or “rejected” given to each hypothesized pathway based on the evidence.

Several strategies will ensure the fidelity and credibility of the interview data, such as using multiple sources of data; creating a chain of evidence that documents all elements of the study database; having both broader research team members and interview participants participate in the triangulation analysis and the return of findings (construct and external validity); examining points of convergence and divergence within and among the data set (internal validity through cross-comparative analyses); and having a stepped analysis process with an initial independent review of the data by 2 reviewers who then meet to reach consensus around the common themes.

### Step 3: Consolidating Program Theory

#### Data Integration and Factor Calibration

We plan to triangulate information from realist interviews, intervention usage data, patient-reported outcomes, and a demographic survey as outlined in Table 2. Patient reported outcome and demographic information will be collected through RCT baseline and follow-up surveys. Intervention usage data will be collected by our third-party collaborator, Memotext, which delivers and manages the RCT intervention. Memotext’s system logs all incoming and outgoing messages, and they will be sharing them with the research team periodically.

We will create a data set in which each interview participant is treated as a unique case and each CIMO element is included as a variable (as defined in the initial program theory outlined in Figure 2). Where variables are continuous (eg, self-efficacy, negative emotions, transition readiness, and blood glucose), we will include them directly. Where variables are qualitative (eg, knowledge and skills, outcome expectancies, behavioral cueing), we will convert them to data-driven categorical variables that will be defined by the research team and reported in the final manuscript.

**Table 2:** Data collection plan for the realist evaluation (new variables may derive from the realist interviews).

Categories and construct			Data source		Measure		Time of measurement (months)
**Context (individual level)**							
	Behavior determinants and contextual factors except demographics	Interview		Self-reported influences on behavior and engagement		Time of interview	
	Demographics	Demographic questionnaire		Gender, ethnicity, insurance type, etc.		0	
**Intervention components**			Intervention usage data and interview		Number of messages sent by KiT chatbot and how the participant perceives each component		
	T1D^a^ self-management information and suggestions					2, 3, 5, 6	
	Stress management strategies					1	
	Transition support information					4	
	Problem-solving support					1, 2, 3, 4, 5, 6	
	Transition reminders					1, 2, 3, 4, 5, 6	
**Intervention features**			Interview		How the participant perceives each feature		Time of interview
	Credible sources						
	Personalization						
	Real-time interactivity						
	User-friendly message tone						
	Diverse forms of information						
**Mechanisms of action targeted by intervention**			Interview		Self-reported behavior changes and perceived impact on targeted mechanisms of action		Time of interview
	Knowledge and skills						
	Outcome expectancies						
	Behavioral cueing						
	Perceived ease of use						
	Perceived usefulness						
**Proximal outcomes **							
	User engagement^b^	Intervention usage data		Response rate to question prompts		1, 2, 3, 4, 5, 6	
	Self-efficacy^b^ and negative emotions^b^	Patient-reported outcomes		SEDM^c^ and BDA Stigma Subscale^d^		0, 6	
**Distal outcomes**			Patient-reported outcomes				0, 6
	Transition readiness			READDY^e^			
	Blood glucose			Self-reported HbA_1c_^f^			
	Self-blood glucose monitoring			Sensor use and additional measures of glycemia for glucose sensor user: whether the participant has been using a sensor for over 70% of the time during the past 14 days at the time of the survey.			

^a^T1D: type 1 diabetes.

^b^Factors will be cross validated using interview data.

^c^Stanford Self-Efficacy for Diabetes Management.

^d^Barriers to Diabetes Adherence in Adolescence Questionnaire Stigma Subscale.

^e^Readiness of Emerging Adults With Diabetes Diagnosed in Youth.

^f^HbA_1c_: hemoglobin A_1c_.

#### Data Analysis

We will conduct CNA using the R package “cna” to consolidate the initial program theory using a bottom-up, data-driven approach [85]. In order to identify the crucial difference-makers of outcomes from a large number of candidate-influencing factors (ie, context, intervention, and mechanism), we will screen the exogenous factors of each outcome before developing CNA models to demonstrate the causal relationships among them.

To achieve factor reduction, we will begin with exploratory data analysis using a routine that operates within the same regularity framework as CNA. Specifically, we will apply the “minimally sufficient condition” (msc) function in the “cna” package to search across the entire data set (ie, all variables and all cases) at once to identify factors with the strongest connections to target outcomes, following the process outlined in Miech et al [86]. We will perform separate analyses in order to identify the minimally sufficient conditions for the presence of desired outcomes (ie, engagement and transition readiness) and those for the absence of desired outcomes, respectively [52,78,79]. For each outcome condition, we will run the “msc” routine 5 times at consistency thresholds of 0.95, 0.90, 0.85, 0.80, and 0.75, examining all 1-, 2-, and 3-condition configurations that meet consistency requirements; have the highest coverage score for their complexity level; and align with theory, background knowledge, case familiarity, and logic [78,87]. Through this process, we will identify a subset of factors to use in the subsequent modeling phase.

In the modeling phase, we will iteratively develop preliminary and final models based on the following criteria: overall model consistency of 0.80 or greater; overall model coverage of 0.80 or greater; and aligns with theory, experience, background knowledge, and logic. Since CNA analyzes data in a bottom-up manner, such analysis will provide empirical findings that can be used to consolidate the initial program theory.

### Ethics Approval

Ethics approval for this study was obtained from the Trillium Health Partners Research Ethics Board (ID: 1086). Ethics for the RCT were obtained from Clinical Trials Ontario through the Hospital for Sick Children Research Ethics Board (Project ID: 3986). The registration number for the larger RCT is NCT05434754.

The participants for this realist evaluation will provide their written informed consent at the time of RCT enrollment. The realist evaluation research team will reach out to consenting RCT participants with a web-based survey link and letter of information with the intention of recruiting and scheduling an interview.

This letter informs participants of their privacy and confidentiality protections, specifically that transcripts will be anonymized and that direct quotes used in reports or publications will not contain any information that could be used to identify them. A deidentified file with the study ID and associated participant information (ie, demographic information, transcript number, intervention usage data, and RCT patient reported outcome data) will be used to organize participant information and will only be accessible by the study coordinator. Only deidentified data will be used for subsequent analyses.

Participants will receive a CAD $25 (US $18.50) electronic gift card for completing the interview.

## Results

The KiT RCT commenced in January 2023. As of May 17, 2023, a total of 36 participants had been enrolled in the RCT. Of the 15 participants who have been assigned to the intervention arm, 13 consented to realist evaluation. Participant enrollment for this realist evaluation was anticipated to start in July 2023 and continue until we reach thematic saturation, or all interested participants have been interviewed. The study findings are planned to be disseminated through peer-reviewed publications and conference presentations in 2024.

## Discussion

### Project Findings and Significance

It is widely agreed that digital health technologies have considerable potential to facilitate diabetes self-management and that user engagement is central to whether or not there is an impact [56]. This study will address several gaps in existing literature, including the identification of which specific features of a digital health intervention facilitate sustained engagement, which threshold level of user engagement leads to desired outcomes, and what individual-level contextual factors facilitate or hinder the implementation process [56,88]. This is a novel application of a realist evaluation to explore nuanced relationships in the context of digital health interventions and T1D health care transitions. It may contribute to knowledge from practical and methodological perspectives, respectively.

While implementation strategies are increasingly informed by implementation science, evidence-based selection of combinations of strategies is often lacking [89]. This realist evaluation is conducted in parallel alongside the RCT implementation, which will enable us to inform real-time refinement of KiT by identifying the features that contribute to desired outcomes and demonstrating the mechanisms of action in real-world settings. Further, this contextualized understanding of whether and how KiT leads to change prioritizes the usefulness of information for decision-making by identifying enablers of and constraints on its delivery across a range of settings [37]. The findings may also shed light on the design, implementation, and evaluation of future digital health interventions that aim to improve transition experiences.

This study will synthesize multiple data sources (ie, qualitative interviews, patient-reported outcomes, and intervention usage data) to enable rich complementary insights [90]. Moreover, this study uses CNA, a novel analytical approach underpinned by Boolean algebra. This approach systematically identifies a “minimal theory,” that is, the crucial combinations of factors linked to target outcomes [89]. By incorporating CNA, we will be able to evaluate empirically the theory-driven configurational models and refine the initial program theory as needed.

### Limitations

First, we are not able to quantify user’s engagement relating to informational content (eg, active days of engagement for a specific module [30]) due to intervention constraints, limiting our ability to understand the value of specific educational elements. Moreover, due to the limited number of interactive SMS text messages, we are not able to measure the change in user engagement frequency using indicators such as the daily SMS text message response rate [91]. As an exploratory mitigation, we will use self-report data gathered by semistructured interviews. We will also use the available intervention usage data to create a composite variable (ie, response rate at 3 months) as a proxy to measure the general level of user engagement. Future studies may benefit from collecting user engagement data at the level of each intervention component and increasing the number of web-based texts to facilitate a more accurate measurement of user engagement. Second, we only consider individual-level contextual factors due to the study objective (ie, identifying target populations for the digital health intervention) and data availability. The omission of setting-level factors (eg, access to health care system, social norms) may limit our ability to comprehensively understand the complex conditions that influence emerging adults’ sustained engagement and health care transition experiences and therefore limit the transferability of our findings under some circumstances [55]. Third, data collection and analysis will occur in parallel alongside the RCT implementation in order to inform real-time intervention refinement. However, timing the study ahead of RCT end point outcome analysis will limit our ability to understand the impact on end point trial outcomes [90]. This was a conscious decision, as many RCTs show suboptimal results [71,92,93], suggesting a need to focus on upstream outcomes such as engagement as a first step.

### Conclusions

Digital health interventions have emerged as a promising resource to support diabetes self-management capacity among emerging adults living with T1D. However, little is known about what components of these interventions are effective, how they are effective, and for whom they are most effective. Taking KiT as an example, our embedded realist evaluation will address this knowledge gap by using a mixed methods design and focusing on an important but often overlooked upstream outcome—user engagement. Besides informing real-time intervention refinements, the knowledge gained from this study may shed light on the design, implementation, and evaluation of future digital health interventions that aim to improve transition experiences.

## Appendix

Multimedia Appendix 1 KiT if-then logic.

Multimedia Appendix 2 Interview guides.

## Abbreviations

CIMO: context-intervention-mechanism-outcome
CMO: context-mechanism-outcome
CNA: coincidence analysis
COM-B: capability, opportunity, motivation, and behavior
DBCI: digital behavioral changing intervention
HAPA: health action process approach
HbA_1c_: hemoglobin A_1c_
KiT: Keeping in Touch
msc: minimally sufficient condition
RCT: randomized controlled trial
READDY: Readiness of Emerging Adults With Diabetes Diagnosed in Youth
T1D: type 1 diabetes
TAM: technology acceptance model

## Data Availability

The data set of this study will be available from the corresponding author on reasonable request.
