# Supplementary material for: Understanding Whether and How a Digital Health Intervention Improves Transition Care for Emerging Adults Living With Type 1 Diabetes: Protocol for a Mixed Methods Realist Evaluation
Source: JMIR Res Protoc. 2023 Sep 13;12:e46115. doi: 10.2196/46115 (PMC10534286; doi:10.2196/46115)
Supplement: Multimedia Appendix 1 [file resprot_v12i1e46115_app1.docx]

### **Appendix 1 KiT Programming if-then logic**

## **[Enrollment](#_heading=h.d7rbhpd1uzeo)**

1. MEMOTEXT sends REDCap link with Study ID to the participant (**RC01**)
   1. MEMOTEXT sends reminders **weekly for 2 weeks following the first message** **(RC01R1, RC01R2)** until the user fills in the survey, the participant is removed from getting more messages after **3 weeks time, trigger an email to RC**
   2. MEMOTEXT sends a confirmation message **(RC01C)** once the REDCap survey has been completed
2. (INTERVENTION ONLY) MEMOTEXT Enrollment Survey **(EN03)** to be sent by MEMOTEXT after REDCap confirmation message.
   1. MEMOTEXT sends reminders **every 2 days following the first message** **(EN03R1, EN02R2)** until the user fills in the survey, the participant is removed from getting more messages after **6 days** and an **escalation email** is sent to the RC
3. (INTERVENTION ONLY) After the Enrollment Survey has been completed
   1. Appointment Messages
      1. If Appointment from REDCap: **AP02** + Schedule Appointment Messages
      2. If No Appointment from REDCap: Schedule **AP03 in 3 Weeks**
   2. Start Proactive Content Messages: PK01
   3. Schedule REDCap Survey Messages (see instructions below)

## **Proactive Knowledge Mobilization (Push Knowledge)**

Content: [Proactive Content List](https://docs.google.com/spreadsheets/d/1XPV5eMnZhyrRZM-qhb0-RP7EK6tn1dKonpfdNfCImpI/edit?pli=1#gid=0)

Send educational content at variable times based on user preferences and topics determined by the READDY assessment. Dates are **randomized** throughout the week with a **minimum one day gap** between randomized messages. The number of content items per week and time ranges content will be determined by the MEMOTEXT enrollment survey (1 -3 times a week)

- Proactive content will start immediately - weeks count starts when participant finishes MEMOTEXT preferences survey and the first message is sent after the completion of the MEMOTEXT preferences survey (mtxt.io/kit_enroll) **PK01** and send no response messaging DE01 if no response based on regular rules
  - Users configure monthly topics every **three** months through text messaging mainly to select their optional content
  - Optional topics are leftover from the list of total topics after the **top 4 READDY topics** are removed and **topics do not repeat**
    - READDY topics are scored on a scale (1 low - 5 high) based on responses the less familiar they are with the topic the lowest 4 READDY topics will automatically be added to the year long program
- Every **4 week cycle** with have **one** topic depending on the order pattern of topics which follow a pattern of Fixed, **READDY, and optional (PK01)**

**PENDING ADDITIONAL MESSAGING THAT WILL GO INDEPENDENTLY EVERY 2 MONTHS**

#### Topics

| **Code** | **Fixed Topics** | **Administration Schedule** | **Branching Dependencies** | **# Messages** |
| --- | --- | --- | --- | --- |
| CP | Coping with T1D | Month 1 | First Message Branching | 5-6 |
| CN | Care Navigation | Month 4 | Ontario and Quebec | 7 |
| SD | Sick Day and Ketone Management | Month 7 |  | 10 |
| MI | Medical Insurance | Month 10 | Ontario and Quebec | 8ON/ 7QC |
| **Code** | **Optional/ READDY Topics** | **Administration Schedule** | **Branching Dependencies** |  |
| HG | Hypoglycemia | variable |  | 7 |
| PP | Pumps and programming | variable |  | 5 |
| IA | Insulin adjustments | variable |  | 4 |
| DA | Drugs and alcohol | variable |  | 8 |
| TV | Travel (not in READDY) | variable |  | 8 |
| RT | Driving | variable |  | 5 |
| AS | School/Work Accommodations | variable |  | 10 |
| EX | Exercise | variable |  | 6 |
| NC | Nutrition and carbohydrates | variable |  | 10 |
| SH | Sexual health | variable | Female and Male | 4M/6F |

Number of Messages a Week for Limited Content

Some of the content buckets do not have enough content to fill preferred frequency, the wording of the frequency has been adjusted to dictate a frequency of up to a certain number.

| **# of total messages** | **1 msg/week** | **2 msgs/week** | **3 msgs/week** |
| --- | --- | --- | --- |
| **4** | 1,1,1,1 | ← | ← |
| **5** |  | 2,1,1,1 | ← |
| **6** |  | 2,2,1,1 | ← |
| **7** |  | 2,2,2,1 | ← |
| **8** |  | 2,2,2,2 | ← |
| **9** |  |  | 3,2,2,2 |
| **10** |  |  | 3,3,2,2 |
| **11** |  |  | 3,3,3,2 |
| **12** |  |  | 3,3,3,3 |

*Initiating Proactive Knowledge Messages*

1. MEMOTEXT Enrollment Survey
2. Send **PK01** to collect optional content
   1. If response, populate PK02 with options calculated from the REDCap Baseline and Fixed topic list
      1. Options = optional READDY topics - bottom 4 READDY topics = 6 optional topics to choose from
      2. remove sexual health if they opted out of sexual health from both READDY options and optional content
      3. remove pump bucket if they have indicated that they use injections in the REDCap survey
   2. No response, send no response message (DE01) after **2 hours** if still no response after **1 day, randomize optional content** and send PK03
3. Start Coping Bucket Messaging
   1. at the preferred time slot send CP00 and CP01 (DE01 if needed)
      1. schedule following messages based on that response
   2. based on the number of days left in that week will determine if they start the content that week or the week after
      1. 2/3 msg per week start next week if less than 3 days left in the week (remember there has to be a day gap between content and coping messages only have max 6 messages so max 2 messages will go out the first week)
      2. weeks also start on a MONDAY

*Continuing 3 Cycle Pattern*

1. **2 weeks** before the 4th,7th,and 10th cycle send PK01 on a day that does not have any proactive content or reminders
   1. Note the list of topics will not include topics in PK01 that were previously picked so the list of optional content will get smaller as they progress through the program
   2. No response, send no response message (DE01) after **2 hours** if still no response after **1 week, randomize optional content** and send PK3

*Sending Messages*

Different buckets may have branching depending on **location** or **gender** of the participant.

- First message is scheduled send a topic intro (usually the 00 message) with the first content message (01) comes together
- randomization of day of the week, ensure minimum a day gap between content messages
- all messages are ordered

*Coping Keyword Trigger*

COPING is going to be added as a keyword to be recognized AFTER the first 4 week cycle so they can get a response to coping messages whenever needed.

1. Type COPING (only after the first month)
   1. if text COPING in the first 4 weeks then get CPK02
2. Get CPK01
3. User responds with a letter
4. Send CPK01R which has a randomly selected message from CPKA, CPKB or CPKC depending on the response (this way, if they type the COPING keyword at any time, and respond with the same answer, they won't be getting the same message each time) and add in an extra line with all resources if they would like access to it.

## **Reactive Knowledge Mobilization (Pull Knowledge)**

Ability for users to ask the system a question and to get keyword-matched resource returned

Questions that Match Keyword Database

1. **#question** must be included in the text message in order for the question to be analyzed
   1. Eg. #question what is the best way to adjust my insulin?
   2. if there is no question inputted after the #question send **QU03**
2. search words in their question based on the keywords column in the content sheet
   1. NOTE: all key words in that list should be UNIQUE if there are any over lapping keywords they must be addressed with the client
   2. if no matches are found send **QU04**
3. for keyword that is triggered send a text message
   1. for each of the keywords, If the keyword has additional messages (see column in content sheet) send **QU02**, if it is the only resource send **QU01a/b** (these are two iterations of the message)
4. after **5 minutes** send message asking for feedback (**QU05**) and once they respond send (**QU05R**)

## **Appointment Reminders**

Appointment reminders are a set of reminders that include all messaging related to the appointments such as appointment reminders, test reminders, notes and appointment prep messages.

- Appointment reminders are usually set **one at a time** and the system will actively be collecting information for their **next** appointment
- Appointment details are collected through **mtxt.io/kit_app** for all appointments
- If new appointment details need to be **updated** the user is able to trigger the appointment message by texting **APPOINTMENT** and updating the current appointment with the new details
- Appointment reminders if they are past the last day of the program
  - send different appointment confirmation **(AP13)** instead of the normal confirmation after they have put in the appointment information
  - on the last day send out the exit message **(EX02)** then send out **(AP04)** and also the notes that they have if any **(NT02)**

*Collecting First Appointment Details*

Data collected from REDCap baseline survey that is administered for first appointment reminder setup from REDCap API

1. If scheduled last pediatric AND/OR first adult then schedule reminders **(AP02)** for the upcoming date based on reminder settings entered in the preferences survey (mtxt.io/kit_enroll)
2. If no appointment is scheduled send a reminder **(AP03)** for an appointment after **3 weeks** repeat reminders every **3 weeks** at preferred time

*Missing Information Reminders*

If the option in the appointment indicates they do not know if they have an appointment test/cgm and/or pump data load send a reminder to update the appointment details every **2 weeks** until information has been updated **(AP11)** or until two weeks before the appointment.

*Updating Appointment Details*

Fill in mtxt.io/kit_app and indicate that they want to update an appointment, after they have finished updating all the details send **AP15** and schedule/update reminders for the updates.

*Appointment Reminders and Appointment Checklists*

Appointment reminders are sent on a set interval determined by the users during their enrollment survey (mtxt.io/kit_enroll) during that reminder period KiT will also provide more information on appointments as well as things to note.

- After Survey Response from mtxt.io/kit_app
  - Send Confirmation > **AP02**
  - If first adult clinic appointment > **AC02/AC03**
- Before Appointment Preferred Time Reminders
  - Appointment Reminder **(AP04)** (Enrollment Survey Preferences)
  - Appointment Checklist (one day before the appointment on preferred time)
    - Last Pediatric Visit **(AP05)**
    - First Adult Visit **(AP06)**
- Before Appointment Test/Data Reminders **(AP12)**
  - Blood Work/ Urine reminder **2 weeks** before the appointment
  - Other (Custom Answer) reminder **variable** and **multiple** based on mtxt.io/kit_app response to question with app_other_reminder
  - CGM/Pump Data reminder **2 days** before the appointment
- **1 Hour** Before Appointment
  - Final Notes List **(NT03)**
    - after this message is sent clear the notes cache for their next meeting
- **Next Day** After Appointment **at Preferred Time**

*Setting New Appointment*

After each appointment **AP09** will ask for new appointment details if no new appointment is set, send a reminder **(AP01)** after **3 weeks** at their preferred time.

## **Notes List**

Participants are able to keep a list of questions that will be added to a notes list which will be sent to the participant 1 hour before their appointment

Adding Notes

1. type the note they want to add to the list with **#note** (will also accept **#notes**)
2. confirm receipt with NT01 message

Showing Notes + Clearing List

1. **1 hour** before their appointment send a list of questions (NT03)
2. Clear the list of notes after that message is sent and all new messages

Showing Current List of Notes

1. Participant texts **SEE NOTES (will also accept view notes, view note)**
2. Send current list of notes added (NT02) if no notes currently exist (NT06)

## **Settings**

Users are able to adjust preferences through text messaging ST01 which will only allow them to edit one setting at a time. If they would like to change another setting they would have to message setting again and then go through the flow.

*ST01:*

Looks like you're hoping to change some settings! Indicate which feature you're looking to change by texting ONE of the following letters. Please reply with SETTINGS again if you would like to edit another feature:

1. Frequency of informational content
2. Time of messages
3. Language
4. Appointment reminders
5. Pause informational content

**NOTE:** updates to the frequency of messages will change on the following Sunday at midnight any changes made before this time will follow the old frequency

*Pausing Proactive Messages*

As a part of the settings pause proactive content is available for 2 week period at a maximum of 2 times.

- If you need a break, you can pause receiving informational content for the next 2 weeks. You'll still get messages like appointment reminders, (don't want you to miss those!) - and just a heads up, you can only do this a maximum of two times during the 12 months that you're in the study.
  Would you like to take a 2-week pause now? Reply with YES to pause.

## **Disengagement And Error Messaging**

For messages that require replies have a reminder to reply that will bring them back into the message at a bit of a later time. If there is continued disengagement this will trigger feedback message.

#### Message Disengagement Process

1. send the participant a message that reminds them of the previous message after **2 hours** of no response (DE01)

#### Email Messages

| **Situation** | **Message** |
| --- | --- |
| **Wrong Number**  STOP command is received before the user fills in the RedCap survey | Subject: KiT Wrong Number for {study_id}  Dear {rc_name}  We have received a STOP command from user with Study ID {study_id}. This is likely an indication of a wrong number.  Action Items   - Please reach out to the user to confirm phone number - If phone number update is required, please update user information at mtxt.io/kit_rc   Sincerely,  KiT Team |
| **Need Assistance**  EN03R2YES message sent = Enrollment Survey  RC0_R2YES message sent = REDCap Survey | Subject: KiT Assistance Needed for {survey} for {study_id}  Dear {rc_name}  User with study ID {study_id} has indicated that they need assistance with {survey}. Please reach out to the users as soon as possible to assist with the survey.  Sincerely,  KiT Team |
| **No Response REDCap**  After 3 weeks no response to the REDCap survey | Subject: KiT No Response to REDCap Survey for {study_id}  Dear {rc_name}  User with study ID {study_id} has not responded to the REDCap survey for 3 weeks. Please reach out.  Sincerely,  KiT Team |
| **No Response MTXT Survey**  No response to MTXT Survey (EN03) after 6 Days | Subject: KiT No Response to Enrollment Survey for {study_id}  Dear {rc_name}  User with study ID {study_id} has not responded to the enrollment survey for 6 days. Please reach out. Completion of the survey is mandatory to start the program.  Sincerely,  KiT Team |
| **Withdraw From Program**  User texts STOP to the program after they have completed the REDCap survey | Subject: KiT STOP command from {study_id}  Dear {rc_name}  User with study ID {study_id} has sent STOP command to the KiT number and is no longer receiving messages from the program. Please confirm participant withdrawal from the program. If message was sent in error the participant must send a START keyword to get messages again.  Sincerely,  KiT Team |

## **Exit Messaging**

Participants will be on the program for a total of **12 full 4-week cycles**, from when the first proactive program content message is sent.

- Reminder for program end sent at **2 weeks** before the end date (EX01) and final REDCap survey link sent at the same time
  - send the final survey reminder at 1 week for the survey completion
- Notification of program stop sent on the end date during their preferred time (EX02)
  - see outstanding appointment details in the appointment section
- If any messages are sent after the end date send generic message of program message end (EX03)
